# Genome-Wide Responses of Female Fruit Flies Subjected to Divergent Mating Regimes

**DOI:** 10.1371/journal.pone.0068136

**Published:** 2013-06-27

**Authors:** Dave T. Gerrard, Claudia Fricke, Dominic A. Edward, Dylan R. Edwards, Tracey Chapman

**Affiliations:** 1 Faculty of Life Sciences and Faculty of Medical and Human Sciences, University of Manchester, Manchester, United Kingdom; 2 School of Biological Sciences, University of East Anglia, Norwich Research Park, Norwich, United Kingdom; 3 Institute for Evolution and Biodiversity, Westfaelische Wilhelms-University, Muenster, Germany; 4 Mammalian Behaviour & Evolution, Institute of Integrative Biology, University of Liverpool, Leahurst Campus, Neston, United Kingdom; University of Arkansas, United States of America

## Abstract

Elevated rates of mating and reproduction cause decreased female survival and lifetime reproductive success across a wide range of taxa from flies to humans. These costs are fundamentally important to the evolution of life histories. Here we investigate the potential mechanistic basis of this classic life history component. We conducted 4 independent replicated experiments in which female *Drosophila melanogaster* were subjected to ‘high’ and ‘low’ mating regimes, resulting in highly significant differences in lifespan. We sampled females for transcriptomic analysis at day 10 of life, before the visible onset of ageing, and used Tiling expression arrays to detect differential gene expression in two body parts (abdomen versus head+thorax). The divergent mating regimes were associated with significant differential expression in a network of genes showing evidence for interactions with *ecdysone receptor*. Preliminary experimental manipulation of two genes in that network with roles in post-transcriptional modification (*CG11486, eyegone*) tended to enhance sensitivity to mating costs. However, the subtle nature of those effects suggests substantial functional redundancy or parallelism in this gene network, which could buffer females against excessive responses. There was also evidence for differential expression in genes involved in germline maintenance, cell proliferation and in gustation / odorant reception. Interestingly, we detected differential expression in three specific genes (*EcR, keap1, lbk1*) and one class of genes (gustation / odorant receptors) with previously reported roles in determining lifespan. Our results suggest that high and low mating regimes that lead to divergence in lifespan are associated with changes in the expression of genes such as reproductive hormones, that influence resource allocation to the germ line, and that may modify post-translational gene expression. This predicts that the correct signalling of nutrient levels to the reproductive system is important for maintaining organismal integrity.

## Introduction

It has long been observed that there are many instances in which elevated rates of mating are costly to females, causing a decrease in female survival and lifetime reproductive success (e.g. [1–7]). This cost of mating in females may represent an evolutionary limit [8] - illustrating how different components of life histories cannot simultaneously be maximised [2–7,9]. There is an additional twist because elevated rates of mating not only cause significant reproductive costs for females, but can simultaneously benefit males [10]. These observations reflect the existence of sexual conflict between males and females that can shape the evolution of reproductive traits for which there are sex-specific optima [11]. Sexual conflict can result in an evolutionary arms race between adaptations in males that, for example, serve to increase mating rate and those in females which serve to decrease remating rate or minimise mating costs [12]. These types of evolutionary conflicts of interest are widespread and can be observed at the level of genes, cells, tissues, life history stages, separate sexes or sex functions, individuals and social groups. Genetic conflicts are important because they drive evolutionary change in a range of traits in the interacting parties. Indeed, conflict is also thought to be key in explaining the high prevalence of chronic diseases such as cancer in humans [13,14]. It is therefore important to understand the underlying basis of life history costs arising from trade-offs.

Trade-offs can occur because of the underlying genetic correlations between the traits involved [2–7]. For example, negative genetic correlations have been documented between early reproduction and lifespan [15,16]. These can be measured as correlated responses to artificial selection or from breeding experiments [17]. The genetic correlations between different life history traits can also often, though not always, be detected using phenotypic manipulations of the relevant traits [2–7,17]. Life history traits involved in trade offs can become ‘uncoupled’ from one another if the genetic correlations underlying them alter because of strong directional selection, or because of changing interactions with the environment (‘gene by environment’ interactions). Alternatively, traits that are usually observed to trade-off with one another may not be involved in obligate negative relationships and can be uncoupled via manipulations of reproductive status and food supply [5,18,19].

In this study we focus on the use of phenotypic manipulations of mating regimes to create divergence in female lifespan and reproductive costs. Several such manipulative studies in *D. melanogaster* females have been performed [20]. This work has sought to identify the factors that explain the decreased survival and reproductive success of females that mate multiply. Separate costs of egg production [21] and of exposure to non-mating males [22] have been identified. However, a significant proportion of female reproductive costs is explained by the effects of male seminal fluid proteins (Sfps) transferred during mating [23]. These molecules alter the female’s physiology and behaviour (e.g. [24]), increasing egg production and reducing willingness to remate [25] thereby increasing a male’s proportion of offspring sired [26]. Each male is unlikely to mate again with his current mate, hence there is selection on Sfp phenotypes to maximise a male’s per mating reproductive success [26] despite the potential costs to females [27]. The reduction in female longevity could result from an acute and specific toxic shock response to Sfps or from accelerated senescence. Alternatively, Sfps can increase the production of juvenile hormone [28], which may increase fecundity but also shorten lifespan [29]. As such, this battle between the sexes may involve the expression of fundamental components of organismal ageing. Of key importance is the interaction of the effects of Sfps with nutrient availability [26,30], with the latter being central to the determination of lifespan [31]. This suggests the hypothesis that mating costs can arise through incorrect signalling of nutrients to the reproductive system.

Previous studies that have sought to identify the source of reproductive costs have focused on the male perspective and tested the effects of male-specific traits such as courtship, male-derived proteins (the Sfps) and their effects on female behaviour. In contrast, virtually nothing is yet known about the processes in females that become disregulated in response to such stimuli, and this is the issue we address here. Several studies have examined the genomic responses of females to one [32,33] or two [34] matings. However, only repeated matings give rise to a significant cost of mating in this species and these studies did not, therefore, assay reproductive costs *per se*.

We examined the genome-wide gene expression changes that occurred in females following the application of divergent mating regimes. We investigated the transcriptional differences in 4-fold replicated groups of females exposed to high and low mating treatments that resulted in significant differences in female lifespan. Cohorts of females were sampled before major mortality effects appeared to avoid the potentially biasing effects of lineage selection, i.e. comparisons of the gene expression profiles of groups in which different numbers of non-random sets of females with lower fitness had already been removed. We tested two different body parts (head+thorax *versus* abdomen) using genome-wide tiling microarrays, and then examined whether there was any evidence that the differentially expressed genes detected interacted with one another. Finally, we experimentally manipulated two of the differentially expressed genes detected, to test for evidence of direct involvement in survival costs of mating.

## Materials and Methods

### Data deposition

The experiment design and both raw and processed expression data have been deposited in ArrayExpress (http://www.ebi.ac.uk/microarray-as/ae/) under accession number E-TABM-800.

### Experimental application of high and low mating regimes

We conducted a 4-fold biologically replicated experiment to test the effect of high and low mating on differential gene expression in females. Following [35] we exposed Dahomey wild-type female *D. melanogaster* to high and low mating regimes. All flies were maintained under 25^°^C on a 12: 12h light: dark light cycle. Females were collected from standard density cultures in which larvae had been reared at a density of 100 per vial containing 8 ml food medium. SY food [36] seeded with *ad libitum* live yeast was used throughout. At the start of each replicate, virgin females were collected at eclosion using ice anaesthesia, and allocated at random to either high or low mating treatments. 60 vials per treatment were set up containing three females and three males each (i.e. 180 females per treatment). In the low mating treatment females were exposed to wild type males for one day in each four-day cycle, and on the other 3 days in 4 to non-mating *poxn* ^70^ males [37], that had previously been backcrossed multiple times into the Dahomey wild-type [35]. The high mating treatment comprised constant access to wild type males but with replacement of new wild type males at the same time as in the low treatment, to control for handling and age of flies. Flies were anaesthetised using CO_2_ to exchange males and during transfer to new food. There are no observable effects of using CO_2_ anaesthesia for transfers throughout life at this frequency on female lifespan [38]. Any effects of CO_2_ anaesthesia on gene expression would have occurred to the same degree in all treatment groups and would therefore not confound the results, unless interacting with one or other of the high and low treatments in an unknown manner. New males were provided on the same four-day cycle until there were fewer than 50 females remaining (including those sampled) in either treatment. The entire experiment was replicated in exactly the same manner 4 times over a 2-month period. We never observed the *poxn* ^70^ males to mate (see also 37), though they courted females frequently. In our previous work we showed that these backcrossed *poxn* ^70^ males court at a rate not significantly different to Dahomey wild type [35]. The inability of *poxn* ^70^ males to mate in our experiments here was re-checked after the fourth replicate experiment. Two sets of vials were set up containing three wild types females and either three wild type males (58 vials) or three *poxn* ^70^ males (60 vials). The adults were allowed to interact for 48 hours then removed. After 7 days, none of the *poxn* ^70^ vials, but all of the wild-type vials, contained larvae or pupae.

We chose this experimental design as it results in a highly repeatable cost of mating, expressed as significantly decreased lifespan and lifetime reproductive success [20,23,35]. The decrease in lifespan seen under this experimental paradigm (intermittent versus continual exposure to males, repeated over 3-4 day cycles throughout life) is not, however, accompanied by differences in age specific reproductive output [20,23,35]. Differences in lifetime productivity therefore result from variation in the length of life. This design therefore permits the effect of manipulating mating frequency on lifespan to be seen directly [20,23]. Survival data were analysed using Log Rank tests, as implemented in the R statistical package ‘surv2sample’.

### Hybridisation to genome tiling microarrays

At day 10, before large differences in female mortality appeared, random samples of 60 females from each treatment were frozen using liquid nitrogen and stored at -80^°^C. We sampled at exactly the same time of day and at exactly the same point in each cycle in each of the 4 biological replicates, to standardise as far as possible the history of the females at the sampling point. The experiments were, however, kept running past the sampling point in order to score lifespan. Thirty of the day 10 sample frozen females from each replicate and treatment were split into Head + Thorax (HT) and Abdomen (AB) samples (dissections were performed over dry-ice). The RNA from these two body parts was extracted separately (mirVana miRNA Isolation Kit, Ambion, Applied Biosystems) and a total of 16 samples (1 time point x 2 body parts x 2 treatments x 4 replicates) were hybridised to individual Affymetrix Tiling 2.0 microarrays (using the Henry Wellcome Polyomics provider, University of Glasgow). We chose to use these two body parts first to try to capture the differential transcriptional responses of behaviour (HT) *versus* reproductive physiology (ABD), and second to limit the non-detection of tissue specific responses due to whole body swamping effects [39]. The 2.0 Tiling array we chose to use features over 3 million 25bp probes spaced, on average, every 39bp. Therefore, unlike traditional microarrays, the Tiling array does not probe for the expression of known or predicted genes. It can, therefore, like RNA sequencing, provide greater resolution than standard arrays for sequences whose functions may be unknown. The intensive sampling of the transcriptome provided by Tiling arrays also permits the detection of differences between specific exons of a gene and between novel transcripts. This technology therefore provides the ease of application of microarray technology but with the increased coverage and resolution of RNA sequencing methods.

### Data analysis: differentially expressed genes

To allow the measurement of differences in gene expression between the high and low mating treatments we chose to use tiling arrays. As described above, we used *D. melanogaster* 2.0 Tiling arrays to test for gene expression differences. As noted, this technology allows tests for differences between alternatively spliced transcripts, though in the data we show here we mainly focussed on currently annotated genes. We assigned 14 tiling probes to each annotated transcript that carried strong or moderate support in Flybase (BDGP Release 5.3, Flybase 2007_02). In total, our custom CDF file contained probe-sets for 13530 transcripts from 10530 genes. The expression data were loaded into ‘R’ using the Bioconductor package Affy [40] and normalised using Robust Multi-array Averaging [41] across all Head-Thorax or all Abdomen tissue sample arrays. Data from probes outside of annotated transcripts were not used in any of the analyses presented here. We searched for differential expression between high and low mating treatments separately using two statistical procedures: (i) mixed model ANOVA (with gene expression = Treatment + Replicate; with treatment as fixed and replicate as a random factors; model implemented in R ‘maanova’ package [42] and (ii) Rank Products [43]. The Rank Products method contrasts the relative positions of genes in ranked lists of expression level between the treatment and control (in this case between the two levels of mating). Strong differences in ranking are correlated with higher fold-changes of expression level [43]. We applied q-values to the ANOVA results to control for false positive and for the Rank Products we used the probability of false positives (pfp) [44]. To test the custom probe sets and analysis, we tested for differential expression between HT and ABD body parts (within the low mating treatment), which should show strong expression differences due to the presence of tissue specific genes. For those genes that were most significantly up-regulated in each body part relative to the other in our data, we assigned each gene to the tissue in which it is most highly expressed according to the independent FlyAtlas dataset [39], Figure S1 shows that the genes we detected as relatively up-regulated in ABD are ovarian genes in FlyAtlas and that our HT genes have greatest expression in the head and brain. Hence this independent test validated our approach.

### Data analysis: Enrichment of gene classes

Using DAVID/EASE [45] we searched for enrichment of functional gene categories amongst those DE genes selected under Rank Products (pfp<0.8). We used the full list of transcripts for which we had mapped probes as the 'background' to provide the programme with an unbiased source gene population. Using individual gene lists for up and down regulated genes in both HT and ABD, and combining these lists, revealed several groups of genes that appeared to have been affected by the level of mating.

### Data analysis: Differentially expressed networks

We examined the potential interactions between the candidate differentially expressed genes, using data from the BIOGRID database [46] and the *Drosophila* Interactions Database (DroID [47]), which included known genetic interactions and high-throughput data from yeast-2-hybrid protein interaction assays. We downloaded all data for *D. melanogaster* from BIOGRID [46]. We filtered the full list for unique pairs of interacting genes and removed self-interactions (e.g. auto-regulation, homodimers) and pairs for which we had not measured expression for both genes. We then calculated a mean differential expression rank for each pair based on each gene’s highest ranking in the RankProd, t-test, or ANOVA analyses, irrespective of direction. Differentially expressed genes with P<10^-4^ (ANOVA) were used to seed interaction networks visualised using the IMbrowser [http://www.droidb.org/IMBrowser.jsp].

### Validation of candidate genes by quantitative PCR

A number of genes of interest were also tested by quantitative PCR (qPCR) using cDNA derived from the same RNA samples as the microarray hybridisation. cDNA was made from 1µg of RNA using Superscript II reverse transcriptase (Invitrogen, Paisley, UK). Primers were designed using the Assay Design Centre (Roche) [https://www.roche-applied-science.com/sis/rtpcr/upl/index.jsp] (Table S4). qPCR was performed on a 7500 Fast Real-Time PCR system (Applied Biosystems, Warrington, UK) using TaqMan Master Mix with the Universal Probe Library (UPL)-probe corresponding to each gene (Roche Applied Science, Burgess Hill, UK). All four biological replicate samples were used. The resulting concentrations were scaled by the sample concentration of *Alpha-tubulin 84B* (CG1913; FBgn0003884). We chose alpha-tubulin as our control after searching multiple datasets for ‘candidate’ control genes (> 30 were surveyed). We investigated both the integrated breadth of tissue expression (via Flyatlas [39]) and constancy of expression through senescence [48]. The latter is an important consideration for any expression study that considers lifespan. CG1913 came out on top as an excellent control on the basis of all these criteria, and also on the basis of its lack of expression variability in our dataset here. For example, in our microarray data, the mean fold change for CG1913 (based on ANOVA) for the abdomen was -0.03 and for the headthorax was -0.02 (both non-significant). Considering the standard deviation of normalised data for all genes, CG1913-RA is also under the 17th percentile for HT samples and under the 5th percentile for AB samples. Given the above we used this highly suitable single control for our analyses. Differential expression was assessed by paired t tests between high and low mating treatments for HT and ABD samples.

### Initial experimental manipulation of differentially expressed genes associated with genomic responses to high and low mating treatments

In two sets of preliminary experiments we then tested the effect on female survival costs of mating of manipulating the differentially expressed genes that we detected. We chose to investigate two genes in the *EcR* network that are associated with post-transcriptional gene expression, namely *CG11486* (*PAN3*) and *eyegone*. We used an EP mis-expression system for CG11486 [49], which causes ubiquitous overexpression of CG11486 when crossed to a line expressing *Gal4* under the control of the *Actin 5C* promoter (Bloomington stock number BL4414: *y* ^1^ *w* ^*^ *; P*{*Act5C-GAL4*} *25FO1/CyO, y* ^+^). This method has been shown to lead to elevated levels of CG11486 transcript [49]. To control separately for the *Gal4* driver and EP mis-expression constructs we crossed each line to *white* ^*Dahomey*^, a line in which the *w* ^1118^ allele had been backcrossed multiple times into the Dahomey wild type background [50]. To test the effect of *eyegone* we used a loss-of-function *eyegone* knock out (Bloomington stock number BL503: *sna* ^*Sco*^ */* ** *CyO* ** *; eyg* ^*1*^) from which we had first removed the *Cy* mutation to remove the need to work with *Curly*-winged flies, which do not fly and tend to become stuck in the food. We also included in each assay a fully wild type Dahomey treatment. Note that each comparison had an internal control not confounded by genotype differences, provided by the test of the same genotype under high and low mating conditions**.**

We followed the experimental design used in the expression analyses described above. To obtain females for the experiments, we combined three pairs of the correct genotypes of parents per vial and allowed the females to oviposit for a period of 24h. The flies were then transferred to a new set of vials the following day and allowed to oviposit for a further 24 hours before being discarded. Vacated vials were incubated for 10 days until offspring started hatching. Dahomey wild type males for the experiments were obtained by culturing larvae at standard density of 100 larvae per vial. Virgin females of the appropriate genotype and wild type males were collected and held in single sex groups of ten. The next day females were randomly assigned to the two mating treatments. Females were held together in groups of three and were exposed to a group of three of the Dahomey wild type males either continuously (high mating) or for one day out of every four (low mating). There were approximately 10 vials of 3 females each per high and low treatments (sample sizes given in Table S1). In the low mating treatment males were removed after the one-day exposure and females were then held alone for the remaining three days of each cycle. Males were replaced every two cycles (8 days) with a new cohort of Dahomey males to keep male reproductive activity high. Groups of flies were switched to new vials on day 2 of each cycle and then remained in that vial for the last three days of each four day cycle, before transferring to a new vial on day 1 of the next. At each transfer males and females were shuffled between different vials within treatments to minimise vial effects. On transfer days densities were adjusted by combining across vials in which females had died, to keep the overall densities constant and the sex ratio at 1:1. Female survival was scored daily, and survival data were analysed using Cox Regression analysis implemented in R.

## Results and Discussion

### Detection of differential gene expression associated with divergent mating regimes

**Figure 1:**
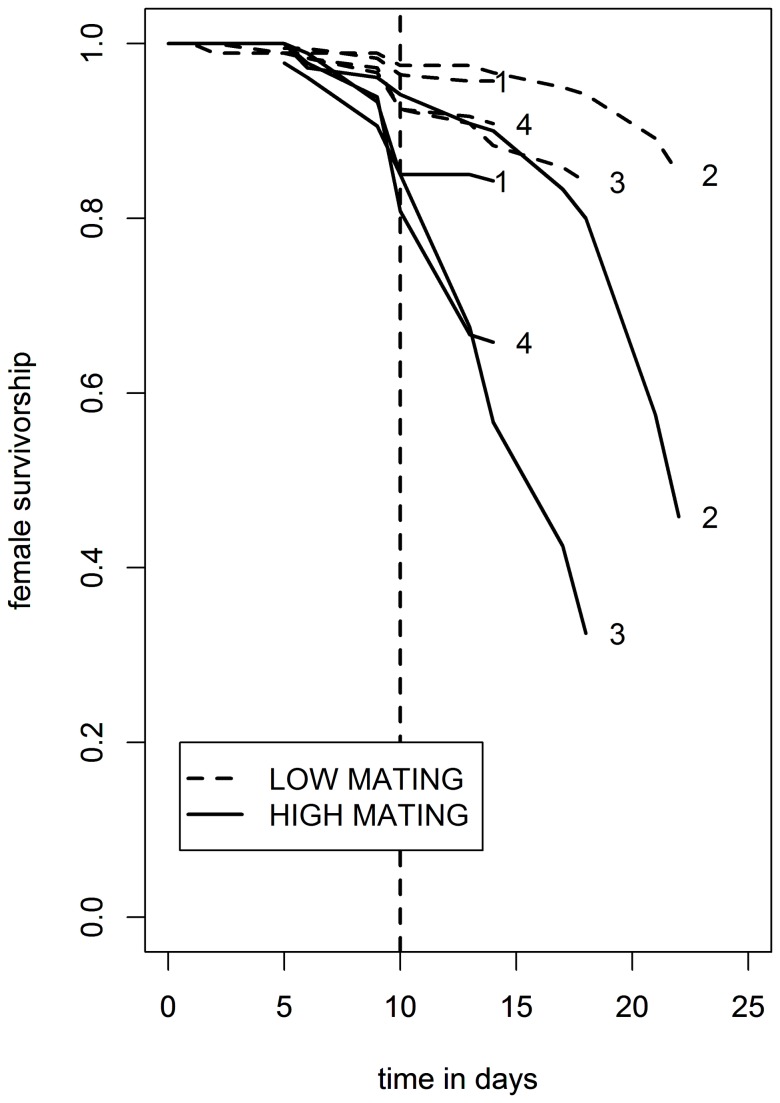
Survival curves for replicate biological experiments 1-4 against time in days for females held under high and low mating treatments. Survival curves are all drawn on the same axes for convenience, but were conducted under identical conditions over a period of 2 months. Samples of 40-60 females were drawn at random from both treatments from all 4 experiments at day 10 (horizontal dotted line). 30 of these females from each sample were then used for RNA extraction and mRNA profiling.

The implementation of the high and low mating treatments resulted in significant female reproductive costs: there was a replicated significant decrease in lifespan caused by elevated mating rate (Figure 1, Log Rank test P values: P=0.045 for replicate 1 and P<1 x 10^-16^ for replicates 2-4; complete survival data and sample sizes are shown in Table S2). Even though these were replicate experiments conducted using identical methods, there was some divergence in the survival patterns across the 4 replicates before the timing of sampling at day 10. This could have had a conservative effect, making the detection of differential expression in some genes more difficult. We did not follow the non-sampled females throughout life, but maintained them for long enough to confirm that we were seeing the expected differences in survival. As described above, the survival data did indeed confirm the expected divergence in lifespan. They were not, however, designed to represent the demography of the females over their whole lifetimes. The aim was instead to check that we were seeing the expected survival difference and to sample females early in the lifespan at day 10 to detect gene expression differences associated with that later survival difference.

RNA was extracted from females on day 10 of each replicate experiment and hybridised to *Drosophila* whole genome Tiling 2.0 arrays (Affymetrix). Separate RNA extractions from Abdomen (ABD) and Head+Thorax (HT) were hybridised to separate microarrays. To compare high and low mating treatments, we used a custom probe-set design, mapping probes to existing gene annotations for 13530 transcripts from 10530 genes (14 evenly spaced probes per transcript). Array analysis was conducted using ‘R’ (see Materials and Methods above). After normalisation for signal intensity across arrays, we tested for differential expression using two statistical procedures: mixed model ANOVA for microarrays (maanova) [42] and Rank Products (RankProd) [43]. Our approach was validated by successfully matching highly expressed HT vs ABD transcripts with known tissue specific expression patterns (Figure S1).

The fold-changes between treatments (high/low mating) were, as expected, more subtle than differences between tissues (Table 1, Table S3). Using a pfp of less than 5%, approximately 100 genes in total (Table S1) were differentially expressed across tissues and treatments (compared to ~2400 genes in the ABD versus HT tissue comparison). Reassuringly, there was overlap between the RankProd and ANOVA methods (Table 1, Table S3). Gene-wise significance was determined with an F-test implemented in the R package maanova (version 1.4.1, option 'Fs') using a Bayesian variance estimate [51]. Of the 82 genes significant under RankProd at pfp<0.05, 80 had Fs (ANOVA) significance values below 0.05 and 60 were below 0.01. Of the top 100 genes found using RankProd, 32 were also in the 100 most significant ANOVA results. Comparing the top 600 from each method, the overlap was 200 genes, again about a third. Notable among the differentially expressed genes detected in the top 10 lists (Table 1) were those involved in *Ecdysone receptor* (*EcR*) signalling (e.g. *eyegone*, *kokopelli*, *CG13373*, *CG11486*) and among the extended lists of differentially expressed genes (Table S3) those involved in olfaction and gustation, sugar transportation and ion channel signalling (see below).

**Table 1:** Top ten differentially expressed (DE) Abdomen (ABD) and HeadThorax (HT) genes for females subjected to high and low mating treatments (Rank Products analysis).

**Tissue / direction of DE**	**Transcript**	**Name/Symbol**	**Fold Change**	**pfp**	***Fs* (ANOVA)**
ABD down	CG32452-RA	CG32452	0.1498	0	0.0001
	CG14779-RA	Pickle	0.1947	0	0.0004
	CG3082-RA	Lethal (2) k09913	0.1554	0	0.0005
	CG10200-RA	CG10200	0.3341	0.004	0.0032
	CG3321-RA	CG3321	0.3368	0.005	0.0050
	CG31012-RA	CG31012	0.3392	0.005	0.0099
	CG32490-RA	Complexin	0.3281	0.0057	0.0039
	CG33861-RA	His1: CG33861	0.4522	0.0344	0.0049
	CG1116-RB	CG1116	0.458	0.0387	0.0614
	CG17800-RV	Down syndrome cell adhesion molecule	0.4342	0.0393	0.0076
ABD up	CG10488-RA	eyegone	5.6291	0	0.0002
	CG14617-RC	CG14617	6.5655	0	0.0003
	CG10365-RC	CG10365	5.6804	0	0.0004
	CG10188-RA	CG10188	4.2421	0	0.0005
	**CG3962-RA**	**Keap1**	4.581	0	0.0013
	CG32490-RK	Complexin	3.2404	0.0037	0.0009
	CG4881-RA	Spalt-related	3.3433	0.0043	0.0027
	CG11486-RC	CG11486	3.3978	0.005	0.0004
	**CG9802-RA**	**Chromosome-associated protein**	3.3562	0.005	0.0032
	CG1712-RA	Gustatory receptor 43a	2.9704	0.0056	0.0219
HT down	**CG9802-RA**	**Chromosome-associated protein**	0.1726	0	6.34E-05
	CG4949-RA	CG4949	0.2234	0	0.0005
	CG9147-RA	CG9147	0.1327	0	0.0007
	CG8502-RA	Cuticular protein 49Ac	0.2109	0	0.0008
	CG6866-RB	Loquacious	0.2186	0	0.0020
	CG32813-RB	CG32813	0.3025	0.0033	0.0024
	CG8068-RC	Suppressor of variegation 2-10	0.258	0.0043	0.0066
	CG13373-RA	CG13373	0.3179	0.0063	0.0008
	**CG3962-RA**	**Keap1**	0.3762	0.0133	0.0150
	CG6921-RA	CG6921	0.3808	0.0138	0.0020
HT up	CG1691-RA	IGF-II mRNA-binding protein	4.5009	0	0.0011
	CG31232-RD	Kokopelli	3.2792	0.01	0.0020
	CG8009-RA	CG8009	4.2475	0.01	0.0023
	CG6030-RA	ATP synthase, subunit d	3.7959	0.01	0.0038
	CG1891-RA	Saxophone	3.7027	0.0125	0.0067
	CG12223-RB	Dorsal switch protein 1	2.6109	0.02	0.0341
	CG9248-RA	CG9248	3.5546	0.0288	0.0074
	CG2368-RF	Pipsqueak	2.4868	0.0329	0.0257
	CG6563-RA	Arginine methyltransferase 3	2.7333	0.0392	0.0146
	CG15920-RA	Resilin	2.3385	0.04	0.0038

Genes in **bold** appear in more than 1 top 10 list. See Table S1 for the full gene list.

**Figure 2:**
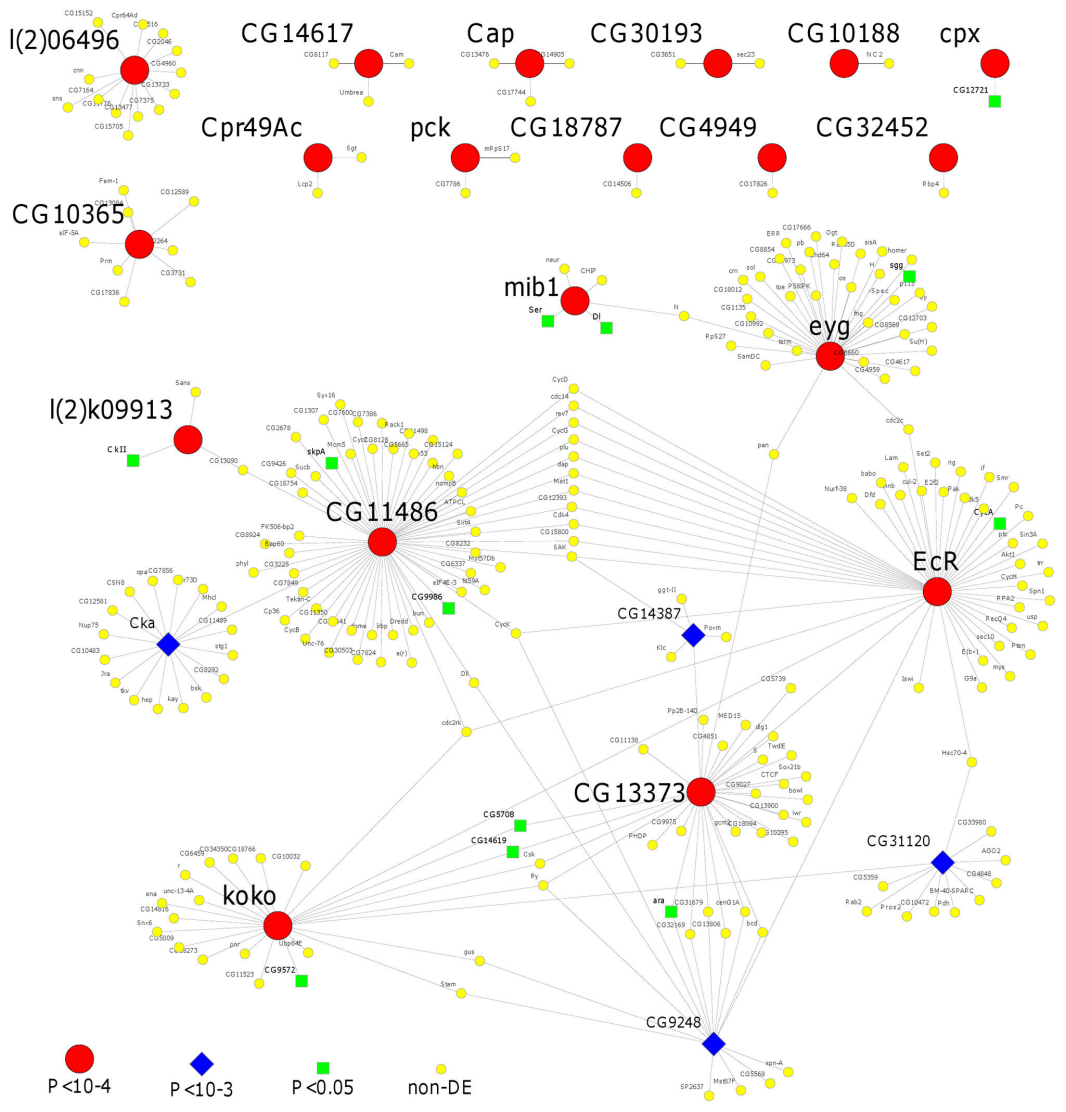
Interaction networks for the most significantly differentially expressed genes resulting from the expression of female mating costs. The interaction network was seeded with the most significant differentially expressed genes under ANOVA P<10^-4^ (red squares). The interactions shown include known genetic interactions and *in vitro* interactions (e.g. yeast-2-hybrid data) but not orthologous predictions from other species. Secondary interactions are not shown unless they include genes that were also differentially expressed at P<10^-3^ (blue diamonds). The network was assembled using the IM Browser at http://www.droidb.org/IMBrowser.jsp and drawn with the aid of Cytoscape 2.6.2 [90]. ‘non-DE’ = non differentially expressed between high and low treatments.

We validated differential gene expression for genes of interest using quantitative PCR (qPCR). We conducted TaqMan assays on all 4 replicates of the same biological RNA samples used to hybridise to the arrays. We chose key genes for which informative Taqman probes were available, from the interaction network (*EcR, koko*; Figure 2) as well as other genes showing differential expression (*CG14617, Cap*) in both tissue types or with plausible links to reproductive regulation and cell proliferation (*Loq, Pickel*) (Table S4). In ABD samples, *EcR, koko* and *CG14617* were all significantly differentially expressed between high and low mating treatments in the direction indicated by the microarray analysis (Figure S2, Table S5). Differential expression in *EcR*, however, applied to transcript A only (see discussion below). The assays for *koko* and *Loq* in the HT did not give evidence for significant differential expression, although *Cap* expression showed a non-significant (P=0.12) difference in the predicted direction (Figure S2, Table S5). Overall the qPCR tests satisfactorily validated the differential gene expression in the chosen genes.

### Differentially expressed genes – global patterns

#### (i) Functional categories

We used the online DAVID/EASE tool to search for enrichment of functional categories of genes selected under Rank Products, using a liberal pfp<0.8 (Table 2). We investigated individual gene lists (e.g. up-regulated in high mating treatment in ABD) as well as combined lists (e.g. all differentially expressed genes at pfp<0.8 irrespective of direction or tissue, Table S6). Where a list specific to one tissue/differential expression-mode showed strong enrichment (>2.0), this was mostly reiterated in the combined lists, i.e. functional categories were generally not limited to one tissue or direction of change. An exception was the enrichment for immune related genes, which occurred only amongst down-regulated genes in HT under high mating (Table 2). Enriched functional categories across all treatments for 1457 transcripts with pfp<0.8 were also analysed (Table S6). Consistent with subtle overall differential expression, the number of genes contributing to each enrichment was modest. Olfactory and gustatory receptors were enriched in HT and ABD samples independently.

**Table 2:** Enriched functional categories in ABD and HT of females subjected to high and low mating treatments, using the online DAVID/EASE functional classification.

**Expression Change**	**Functional Category**	**Enrichment**	**Generated by:**
HT Down	Immune response	2.26	6 genes
	Sugar transporters	1.41	8 genes
	Gustatory & odorant	1.26	8 genes
HT Up	ATP binding/Kinase	1.3	25 genes
ABD Down	Ion channel activity	1.31	6 genes
ABD Up	Gustatory & odorant	2.26	21 genes
	Ion channel activity	1.98	16 genes

#### (ii) Interactions and networks

Potential interactions between the candidate differentially expressed genes were examined using data from the BIOGRID database and *Drosophila* Interactions Database (DroID), which include known genetic interactions and high-throughput data from yeast-2-hybrid protein interaction assays. The results from the BIOGRID analysis revealed 29 interactions between *EcR* and another gene present in our dataset. Of these, ten interactions featured *EcR* and another gene ranked in the top 500 (from 18759 unique interactions where both genes were scored in our dataset). The direct interactors of *EcR* were over-represented near the top of our list of genes showing altered expression under high mating. Furthermore, interactors of *EcR* interacted multiply with other differentially expressed genes with low *P* values under the ANOVA analysis (Figure 2). *EcR* itself did not appear in the lists of genes from the RankProd analysis (Table S3) because its fold change was modest. However, the gene expression differences in *EcR* were consistent across all 4 replicate experiments, giving a highly significant result from the ANOVA analysis (Fs P value=0.0007). Significant differential expression was confirmed also in the qPCR validation (Table S5 Figure S2). Many of the most significantly differentially expressed genes had no or very few interactions and did not show interactions with other differentially expressed genes (satellite interactions shown in Figure 2). However, 7 of the differentially expressed genes interacted directly or through a single intermediate (Figure 2). This single gene network included *EcR*, *Kokopelli* (*koko*), *eyegone* (*eyg*), *CG13373*, *CG11486*, *I(2) k09913*, *mib1* all at P<10^-4^ and also *CG9248*, *CG14387*, *CG31120*, *Cka* at P<10^-3^. Though the anonymous genes have mostly not been ascribed gene ontology (GO) classifications, they are linked to them by the related roles of control of cell-fate, proliferation signalling and cell division to *EcR* and *kokopelli* (see below for further evidence of *EcR* interactions). This strongly-supported interaction network of differentially expressed genes reveals a link between the cost of mating and maintenance of the germline niche [52] and stem cell differentiation through ecdysone signalling. We also tested for interactions of the genes highlighted by DAVID/EASE analysis. Of the 83 genes contributing to the functional enrichment in Table S6, none are involved in potential direct interactions with one another (based on the interaction datasets above).

It is interesting to note that the differentially expressed genes we identified as interacting with *EcR* were not those implicated to date in canonical *EcR* signalling (e.g. *broad, E74, E75* [53]; [54,55];). It could be that differential expression in those canonical components was too small to be detected or was not present because not all components of any pathway are necessarily the key ‘limiting’ factors in the response to divergent mating regimes. It is possible that we have identified a parallel non-canonical *EcR* signaling pathway, or alternatively a pathway in which EcR plays an as yet unknown functional role in determining the response of females to divergent mating regimes. Further work is required to distinguish these possibilities.

### Classes of differentially expressed genes associated with high and low mating regimes

The genomic signature of responses to high and low mating leading to survival mating costs was subtle but significant, and the genes identified were connected by shared functions and direct interactions. A striking result was differential expression in a single network of genes that all interact with *EcR*, suggesting that this network may be a key part of responses associated with survival costs of mating. It is noteworthy that reduced *EcR* signalling has already been identified as important in extending female lifespan [56]. Functional categories identified in the differentially expressed genes were gustatory/odorant, sugar transporters and genes related to ion channel activity. The biological variability across the 4 replicate experiments, even though conditions were thoroughly standardised, suggests that there could also be signatures of mating costs in addition to those we have detected. Expression differences did not overlap significantly with those detected in previous genome-wide studies that investigated the transcriptional response to one or two matings (e.g. [32–34]). Nor did we detect significant overlap with common stress response pathways (e.g. [57]). Taken together, our data suggest that the responses to divergent mating regimes leading to mating costs in females may be mediated, at least in part, through adjustment of mechanisms controlling investment in somatic maintenance and reproduction, as indicated by changes to *EcR* signalling. We explore the potential significance in terms of specific genes and pathways below.

#### (i) Ecdysone Receptor and interacting genes

High levels of ecdysone shorten lifespan, and this effect is ameliorated in *EcR* null mutants in which ecdysone signalling is reduced [56]. However ecdysone signalling is essential during development and is required for continued stem cell proliferation in adults [58]. The finding that *EcR* isoform A was up-regulated with increased mating represents potential evidence of a link between ageing [56] and investment in reproduction in females. This interpretation is also supported by the finding that early germ line ablation leads to extended lifespan [59], see also 60. We detected no differential gene expression in *EcR* B1 in either HT or ABD, which suggests that the ratio of the two isoforms varies, which may be important for function. Larval tissues are known to have both a mixture of discrete and overlapping expression patterns of A and B1 EcR isoforms [61]. A and B1 are both widely expressed in adult tissues at low levels [62], with B1 being required for oogenesis [63]. Future work to delinate the contribution of the different EcR isoforms in adults may yield further insight into functional specificity [55]. We note that our ability to detect different signals of expression in the A and B *EcR* transcripts highlights the utility of Tiling arrays for profiling. Our early sampling (day 10) combined with the potential of *EcR* to interact multiply with other differentially expressed genes suggests that expression changes in *EcR* could be an early signature of mating costs. A mating signal could act to divert resources to reproduction, or could imitate a systemic glut of resources and ‘trick’ the female into provisioning more into reproduction than is optimal. However, it is important to note that our data show day 10 genome-wide responses to the divergent mating regimes applied but not necessarily to the later survival differences *per se*.

Signalling of the nutritional status to the dividing germ line stem cells in the ovary is essential to achieve the correct rate of cell division. However, little is yet known about the mechanisms by which this is achieved [64]. *kokopelli* could be a good candidate for early signatures of mating costs as shown by its response to the divergent mating regimes. Our array data suggested there was significant differential gene expression in *koko* in both HT and ABD, a result validated by qPCR in the ABD (Table S3) but not HT, perhaps because of relatively low transcript levels in the HT overall. Although widely expressed, *koko*, like *EcR*, is required for oogenesis (J.D. Baker and M.J. Kernan, pers. comm). *koko* is a cyclin gene and its effects on oogenesis appear to be mediated through control of cell fate determination. *EcR* and *koko may* therefore interact to maintain oogenesis, though this new hypothesis requires further direct experimental evidence. The data from Figure 2 suggest that both *EcR* and *koko* also interact with *CG11486*, a gene that is discussed in the context of post-transcriptional gene regulation, below.

#### (ii) Gustatory/odorant genes and sugar transporters

We found evidence for enrichment of gustatory/odorant genes, with high mating leading to down regulation in the HT and up regulation in the ABD. This may reflect increased traffic of nutrients in response to higher demands upon the reproductive system. Gustatory/odorant receptors are expressed in sensory sensillae and function in the detection of sugar and bitter tastes, pheromones and CO_2_ [65]. Interestingly, as for *EcR*, a role for gustation / odorant receptors has previously been identified as a determinant of lifespan - with downregulation of gustation / odorant perception (via loss of function of odorant co-factor *Or83b*) leading to increased lifespan [66]. Gustatory/odorant receptors can also be expressed internally [67], however the roles of these types of receptors in internal nutrient signalling are as yet unknown. Gustatory receptor (Gr) *43a* featured in the top 10 up-regulated genes in the ABD, but it is not yet known whether it is involved in external or internal nutrient sensing. The finding of differential expression of nutrient sensing genes is consistent with our hypothesis that costs may arise through incorrect signalling of nutrient levels to the reproductive system.

#### (iii) Immune genes

It has previously been suggested that mating costs could result from the disregulation of the immune system [34]. Several studies have found evidence for connections between reproduction and immunity across insects, mammals and birds (e.g. [68]). Intriguingly, regulatory effects of ecdysone on the expression of immunity genes in insects is also reported [69]. However, our results do not provide strong support for the view that immune genes were strongly differentially expressed in our high and low mating regimes. We detected only a minor signature of differential gene expression in immune genes in the functional analysis of gene categories (Table 2). In addition, immune genes did not feature strongly in the gene lists (Table 1, S1). Differential gene expression in immune genes has been reported in a wide variety of studies of transcriptional responses [32,33,70]. It is as yet unclear how specific or long lived is the response of immune genes to mating, although specific Sfps transferred by males during mating can alter the expression of immune genes [71]. The overall fitness effects of altered immune signalling are also, at present, unclear [68].

#### (iv) Post-transcriptional regulation

Transcript CG11486-RC showed significantly increased expression under high mating in the ABD but decreased in the HT and appeared near the top of both gene lists and in the network of differentially expressed genes interacting with *EcR* (Figure 2). *CG11486* is the likely orthologue of the human gene, ‘*polyA specific ribonuclease* 3’ (*PAN3*), which is involved in the de-adenylation of mRNAs and their subsequent breakdown [49] in a critical first step in the control of gene expression by microRNAs [72]. Previously, Mack et al. [73] showed that the expression of *CG11486* was altered after single matings. We suggest that this transcript may aid in the responsive release or inhibition of other mating-related gene products via post-transcriptional mechanisms.

The *Pax6* homologue *eyegone*, which has multiple roles during development, was another differentially expressed gene in the *EcR* network, showing 5-fold increased expression under high relative to the low mating treatment. *Eyg* is a transcription factor whose role in adults has not yet been well characterised, though it is enriched for expression in both the adult salivary glands and male accessory glands [39]. In general, however, *eyegone* acts as a repressor of gene expression in its target gene [74]. *Loquacious* (*loqs*), which is linked to adult germ-line stem cell maintenance [75], was one of the most strongly downregulated genes in the HT. *loqs* is a key interactor with *dicer-2* in the maturation of short interfering RNAs that, in turn, exert post-transcriptional regulation of many other genes. Together these findings predict that small RNAs may play a role in the expression of mating costs via post-transcriptional gene regulation. This is an interesting topic for future investigation.

#### (v) Apoptosis, cell cycle and cell division genes

An additional aim of the study was to gain insight into mechanisms underlying evolutionary conflicts of interest between the sexes. As such conflicts may also promote proximate mismatches between the regulation of essential processes at the organ and tissue level, they may be important in the development of age-related diseases such as cancer [14]. Control of apoptosis and the cell cycle are intimately linked and are seen as opposing forces in cancer development [76]. It was therefore notable that BIOGRID interactions analysis listed 59 potential direct protein interactors of the CG11486 protein described above, 14 of which were cell cycle control genes. Twelve are also potential interactors with *EcR* (Figure 2), and three (*decapo*, *plutonium* and *Cdk4*) showed evidence of significant differential gene expression (ANOVA P values < 0.05). A functional analysis (DAVID/EASE) on interactors of CG11486 showed strong enrichment for control of cell cycle genes (cyclins and cyclin dependent kinases) suggesting that CG11486 is involved in the regulation of cell growth and division. We did not subject CG11486 to qPCR validation because of its large number of alternative transcripts (~15). However, we did test it experimentally for direct involvement in determining the magnitude of survival costs of mating, see below. We were also interested in *pickle*, which showed evidence of strong down-regulation in the ABD under high mating, because of its links to cell division. *Pickle* is widely expressed and produces a claudin protein that functions in the cohesive interactions of epithelial cells [77]. Such proteins, when disregulated, are associated with cancers in human reproductive tissues [78].

We described differential expression in several genes associated with chromosomal segregation during cell division (Table 1, S1). Transcript CG9802-RA (*Cap*) was ranked 2 in genes down regulated in the HT (>5-fold reduction) and 10 for up-regulated in the ABD (>3-fold increase). The gene has an essential function in sister chromatid association [79] and could contribute to chromosome stability during cell division or function in mature, differentiated cells, for example, in chromosomal control of transcription. Another gene of note was *CG14617*, which showed significant up-regulation under high mating for both HT and ABD. This gene is relatively unstudied but features in several high throughput expression datasets. During development the pattern of *CG14617* expression resembles that of *cyclin A* at stages 13-16 in the mitotically active ventral sensory complex primordium [80]. In adults, the strongest expression is in the reproductive tissues with much reduced, but consistent expression in the head and brain [39]. Combined, these data suggest a role for *CG14617* in cell division, which occurs mostly in reproductive tissues and to some extent in the adult *Drosophila* brain [81].

### Tests for mating costs through manipulation of differentially expressed genes

Direct tests with either loss- or gain-of-function manipulations are a powerful method to test for evidence that the differentially expressed genes identified in this study directly influence the expression of mating costs in females leading to decreased lifespan. Of the specific genes we identified as differentially expressed (Figure 2, Table S3), to our knowledge, only *EcR* [56], *Keap1* [82] and *lkb1* [83] are so far reported to have direct effects on lifespan, as described below.

*EcR* is the best characterised; both males and females heterozygous for a null mutation of *EcR* are reported to show increased lifespan and stress resistance [56]. Increased lifespan associated with lowered ecdysone titres in *EcR* biosynthetic pathway mutants can also be reduced by ecdysone treatment [56]. However, later studies provide partially contradictory findings, and suggest that the effects of *EcR* on lifespan show sex-specificity, with reduced *EcR* levels leading to extended lifespan in males and reduced lifespan in females [84]. Hence the relative levels and sites of expression of *EcR* appear to be critical in determining the magnitude and direction of lifespan effects. The role of *EcR* in the determination of lifespan, male and female fertility are, however, consistent with the findings from our study that *EcR*, and the genes with which it interacts, play central roles in the expression of mating costs. Turning to the other two genes, heterozygotes of a loss-of-function allele of *Keap1*, a gene with important roles in defence against oxidative stress, have extended lifespan in males but not females [82]. Finally, *Lbk1* encodes a kinase that is involved in regulating signalling through the *TOR* pathway during conditions of nutrient stress, and its overexpression can result in a small but significant extension to male but not female lifespan [83].

**Figure 3:**
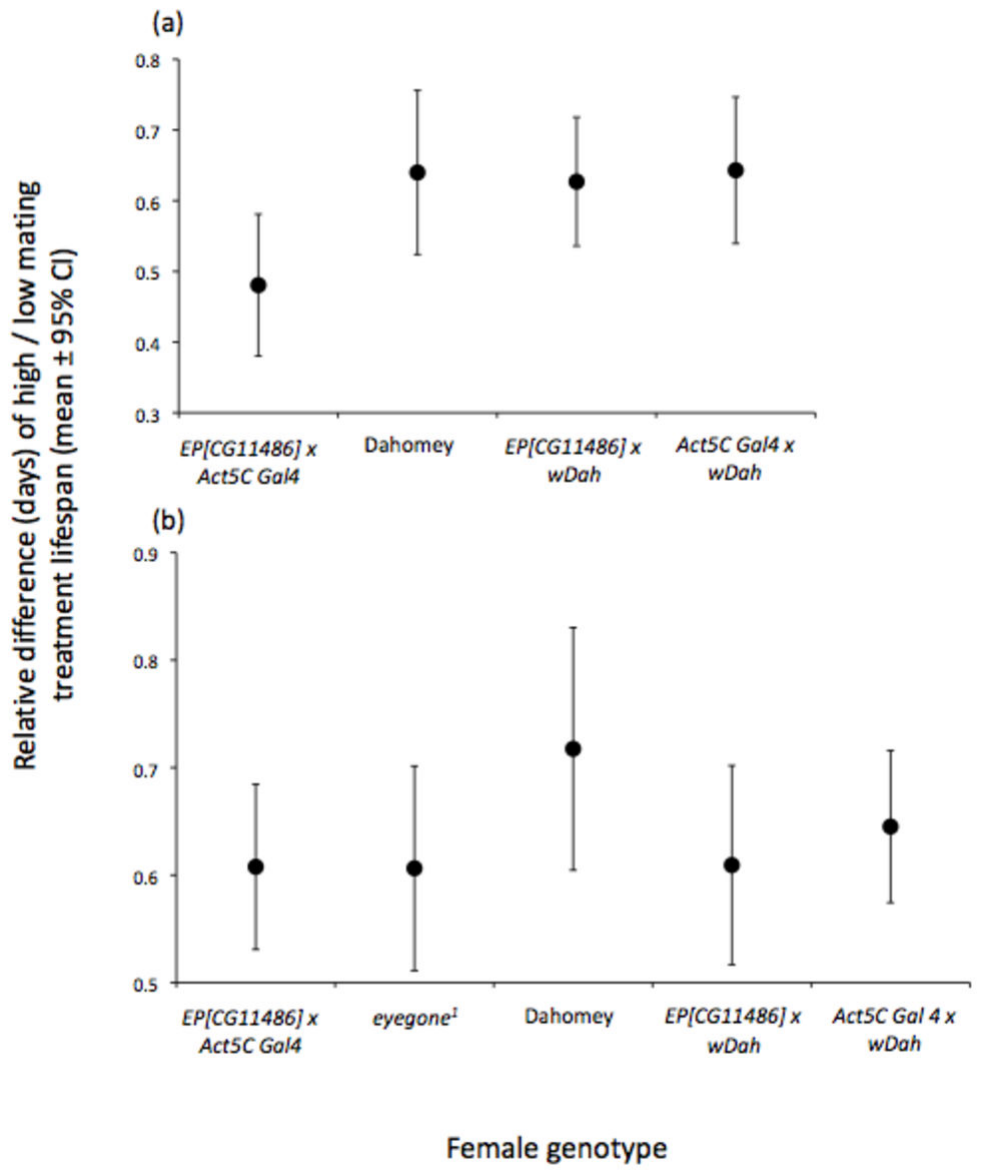
Average relative lifespan (high lifespan divided by low lifespan, mean days ± 95% confidence interval) of high relative to low mating *CG11486* and *eyegone* mutant females. (a) Experiment replicate 1: average relative female lifespan (mean days ± 95% confidence interval) for *EP*[*CG11486*] *x Act5C Gal4* (*CG11486* overexpressing), Dahomey (control), *EP*[*CG11486*] *x wDah* (control), and *Act5C Gal4 x wDah* (control) females. (b) Experiment replicate 2: average relative female lifespan (mean days ± 95% confidence interval) for *EP*[*CG11486*] *x Act5C Gal4* (*CG11486* overexpressing), *eyegone* ^1^ (*eyegone* knockout), Dahomey (control), *EP*[*CG11486*] *x wDah* (control), and *Act5C Gal4 x wDah* (control) females.

None of the genes above has, to date, been tested experimentally for direct involvement in the expression of mating costs. We therefore conducted direct preliminary tests with two genes from the *EcR* network described above. We chose genes central to this network, and those that also allowed us to test the hypothesis that post-transcriptional regulation is important in the response to high and low mating: *CG11486* (*PAN3*) and *eyegone* (for full survival data and sample sizes, see Table S1, S7). In the first replicate we tested the effect on the expression of female mating costs of overexpression of *CG11486* (Figure 3, Figure S3). There were significant effects of female genotype (Cox regression analysis: χ^2^_3_ = 27.19, P<0.001) and of high or low male exposure (χ^2^_1_ = 137.69, P<0.001) but no significant interaction (χ^2^_3_ = 1.76, P=0.62). The average lifespan of *CG11486* overexpressing females overall was significantly shorter than for the average lifespan of the controls (orthogonal contrast, P<0.001, including wild type Dahomey). High mating *CG11486* overexpressing females lived only 50% as long as lower mating females of the same genotype, whereas in the two controls and the Dahomey wild type, high mating females lived relatively longer (65% of the low mating lifespan; Figure 3, Figure S3). Hence overexpression of *CG11486* tended to cause females to express mating costs at a higher, but not significantly higher, level than in controls.

In the second replicate set of experiments we repeated the tests of overexpression of *CG11486* and also tested the magnitude of mating costs in *eyegone* ^1^ knockout females. We detected a significant interaction between the effect of high or low male exposure and female genotype (Cox regression analysis: interaction χ^2^_4_ = 10.64, P=0.031). As above, there were significant effects of female genotype (χ^2^_4_ = 119.88, P<0.001) and of high or low male exposure (χ^2^_1_ = 167.11, P<0.001). Consistent with the first replicate experiment, the overall average lifespan of the *CG11486* overexpressing and *eyegone* lacking females was significantly shorter than for the average lifespan of the controls (P<0.001, including wild type Dahomey). High mating CG11486 overexpressing and *eyegone* ^1^ females lived 60% as long as low mating females of the same genotype. In contrast, the relative lifespan of the high mating controls tended to be longer, though *EP*[*CG11486*] *x wDah* female controls were also relatively short lived in comparison to their low mating counterparts (Figure 3, S3). Overall the significant interaction between high or low male exposure and female genotype suggests there may be variation in female sensitivity to survival costs of mating. However, females overexpressing CG11486 and lacking *eyegone* showed high survival costs of mating in comparison to only two out of the three controls. Hence, as in the first experiment, any effects are subtle. Overall, the results of these initial investigations can only be regarded as preliminary. Further work should be conducted with larger sample sizes and more loci, avoiding the known pitfalls of tests with very short lived mutants [85] and also using other methods (e.g. such as the ‘Geneswitch’ system that could restrict manipulations to the adult stage [86] and provide good internal controls for genetic background [84]. In addition, we note that our knockout *eyegone* ^*1*^ line was not fully backcrossed into the wild type background (only the *Curly* wing marker had been removed). While there was an internal control as there was for each test (high mating versus low mating treatments of the same genotype) we cannot rule out off-target effects of other unknown loci. Future work should therefore focus on tests with lines that have undergone further backcrossing, so that effects of the focal loci targeted can unequivocally be observed.

The lack of substantial effects on the expression of mating costs of direct manipulation of differentially expressed genes in the *EcR* network could be due to several reasons. First, effect sizes may be relatively small, making differences statistically difficult to detect. Note for example that even for moderate effect sizes the Cox regression method and other similar survival analyses are relatively insensitive to interaction effects between survival curves. Second, there could be functional redundancy in this *EcR* network such that the manipulation of individual component genes in it results in less of an overt phenotype than might be expected. Such redundancy in gene networks may provide an effective way in which to buffer the organism against social, sexual and environmental variation. This buffering could therefore protect females against high mating costs. Finally, it is also possible that there are parallel, as opposed to redundant pathways operating, such as is reminiscent of the effects on ageing and survival of many components of dietary restriction [19,31,87] and other aging genes/pathways (such as the insulin and TOR pathways [88,89]). Future tests are now needed with simultaneous manipulation of several genes in the *EcR* network to test these ideas.

## Conclusions

Overall, our main finding was that exposure to elevated mating rates led to differential expression in *EcR* and putative interacting genes, as well as in gustation/odorant and sugar transporter genes. Initial experiments to manipulate two genes (*CG11486, eyegone*) in the network of genes interacting with *EcR* led to only subtle alterations in the magnitude of survival costs of mating expressed by females. This could be because there is no direct link between the response to the mating regimes and the later survival differences. However, the detection of several genes known to have effects on survival argues against this idea. The findings instead suggest the existence of putative functional redundancy or parallelism within the network of differentially expressed genes, which may serve to buffer females against excessive costs. We identified differential expression in two key sets of genes – those that interact with *EcR* and those involved in gustation / odorant perception and signalling. Significant changes to *EcR* signalling suggest that alterations to the germline niche and regulation of oogenesis [52] are associated with divergence in the mating regimes and perhaps the associated effects on lifespan. We suggest that this occurs because *EcR* signalling dictates how resources are channelled to either lifespan versus reproduction. The extended lifespan seen upon altered *EcR* signalling is consistent with this idea [56]. Additional support is provided by our finding that there was also differential expression in gustation and odorant genes, which too are known to be able to extend lifespan upon our data suggest the hypothesis that elevated mating and the associated loss of lifespan are associated with the expression of genes that control how resources are allocated to the germ line. Determining in the future how this disruption is instigated, and whether it predicts a loss of homeostasis in general, will give us significant insight into trade-offs between reproduction and ageing.

## Supporting Information

Figure S1 Validation of the custom probe set analysis method. We analysed the concordance of tissue specificity in the differentially expressed genes detected, by cross-referencing to the Flyatlas [39] of adult gene expression. For each bodypart (Abdomen (ABD) in grey or Head + Thorax (HT) in black), the 100 genes with highest expression level detected in this study are grouped by their tissue of greatest enrichment as listed in the Flyatlas database. tag: thoracoabdominal ganglion; sal. gland: salivary gland. The figure confirms that, as expected, differentially expressed genes from the HT were those listed in Flyatlas as being expressed in brain, head, tag, crop, etc and those in the ABD with those expressed in the ovary.
(TIF)

Figure S2 qPCR analysis of differentially expressed genes (as detected by the arrays) in the Abdomen (ABD) and HeadThorax (HT) body parts of females subjected to high and low mating treatments. Graphs show the normalised relative levels of gene expression (mean high / low normalised expression ± 95% confidence interval) as determined by qPCR assays for high and low mating treatments in biological replicates 1-4 combined for each of the genes shown. The expected ratio is predicted from the direction of putative differential expression observed in the microarrays (>1 represents putatively up-regulated in the high mating group, and <1 putative down-regulation; see also Table S5). The dotted line represents equal expression in the high and low treatments. One tailed t test P values are shown.
(TIFF)

Figure S3 Female survivorship against time in days for *CG11486* and *eyegone* mutant females subjected to high and low mating treatments. Experiment 1 (a)-(d): survivorship of high and low mating females of the following genotypes: (a) *EP*[*CG11486*] *x Act5C Gal4* (*CG11486* overexpressing), (b) *Act5C Gal4 x wDah* (control), (c) *EP*[*CG11486*] *x wDah* (control), and (d) Dahomey (control). Experiment 2 (e)-(i): survivorship of high and low mating females of the following genotypes: (e) *EP*[*CG11486*] *x Act5C Gal4* (*CG11486* overexpressing), (f) *Act5C Gal4 x wDah* (control), (g) *EP*[*CG11486*] *x wDah* (control), (h) Dahomey (control), (i) *eyegone* ^1^ (*eyegone* knockout).
(TIF)

Table S1 **Sample sizes for the initial tests of mating costs in *CG11486* and *eyegone* manipulated females.**
(PDF)

Table S2 Survival data and sample sizes for replicates 1-4 of the experiment to test the effect of exposure to high and low mating treatments on genome-wide gene expression changes in females. (a) Number of females alive per day and timing of sampling for gene expression analysis. (b) Cumulative number of deaths per day. (c) Number of female dead since last censoring. (d) Survivorship (cumulative survival probability). (e) Survivorship differences between treatments (low minus high treatments).
(PDF)

Table S3 All differentially expressed genes in the ABD and HT body parts of high and low mating treatment females. Results are from the RankProd analysis using a pfp<0.05 (see main text for more details). Genes in bold appear in more than one list.
(PDF)

Table S4 Genes and isoforms surveyed by qPCR. Given are PCR primers used for each of the genes tested and the Universal Probe Library probe used in the qPCR analysis.
(PDF)

Table S5 qPCR analysis of candidate DE genes from the microarray analysis. Shown are (a) the fold changes and significance values from the microrarray analysis, and (b) the isoforms tested by qPCR, the fold difference (high / low treatment expression values), the Universal Probe Library (UPL) probe used and finally significance values from 1 tailed paired t-tests (P values < 0.05 are shown in bold). See also Figure S2.
(PDF)

Table S6 **Enriched functional categories resulting from DAVID/EASE analysis of all differentially expressed genes in the ABD and HT body parts of females subjected to high and low mating treatments (see main text for more details)**.
(PDF)

Table S7 **Mean ± 95% confidence intervals of female survival in days in the initial tests of mating costs in *CG11486* and *eyegone* manipulated females.**
(PDF)
