# Supplementary figures and images for: Genome-Wide Responses of Female Fruit Flies Subjected to Divergent Mating Regimes
Source: PLoS One. 2013 Jun 27;8(6):e68136. doi: 10.1371/journal.pone.0068136 (PMC3694895; doi:10.1371/journal.pone.0068136)

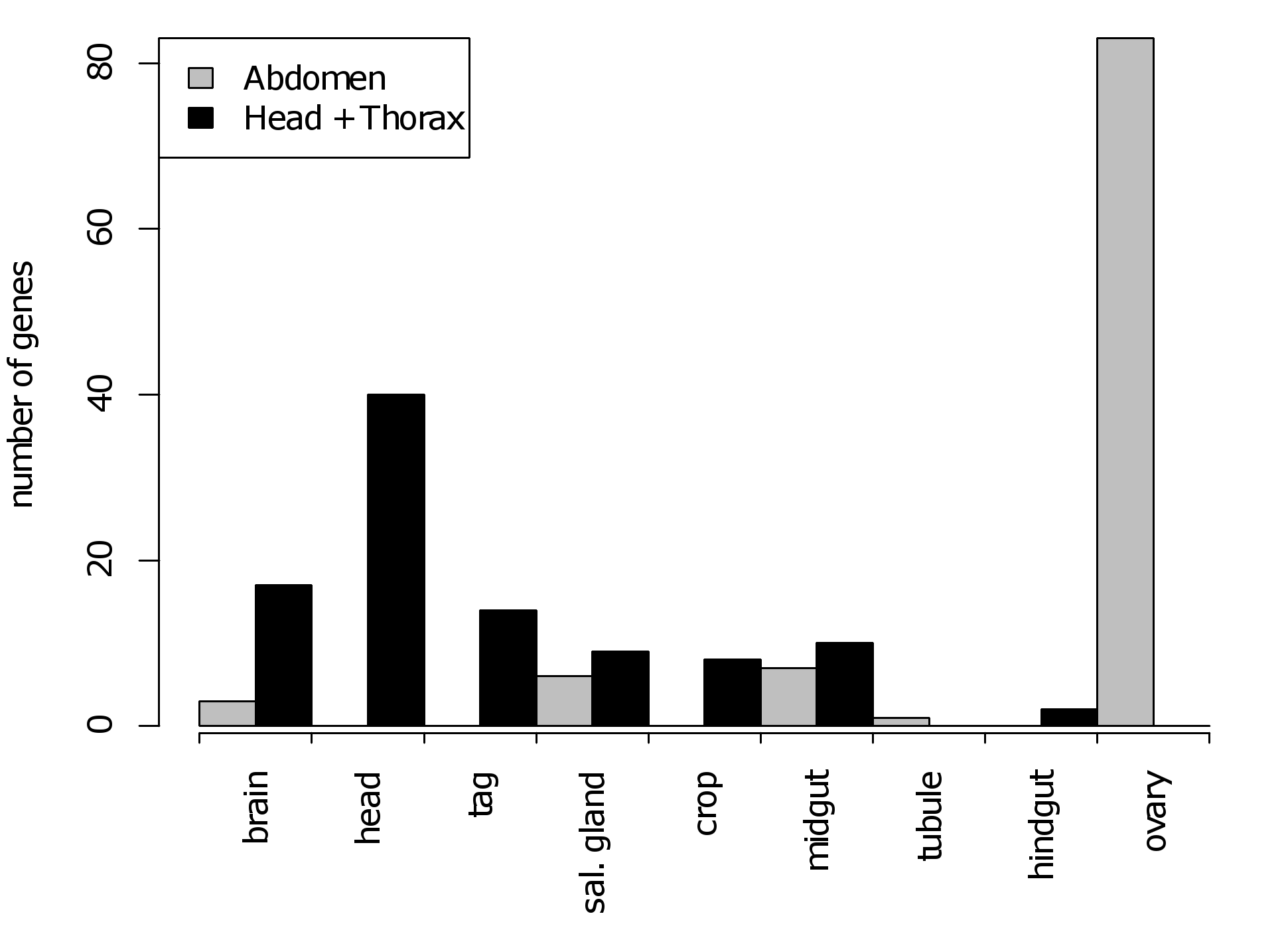

Supplement: Figure S1 — We analysed the concordance of tissue specificity in the differentially expressed genes detected, by cross-referencing to the Flyatlas [39] of adult gene expression. For each bodypart (Abdomen (ABD) in grey or Head + Thorax (HT) in black), the 100 genes with highest expression level detected in this study are grouped by their tissue of greatest enrichment as listed in the Flyatlas database. tag: thoracoabdominal ganglion; sal. gland: salivary gland. The figure confirms that, as expected, differentially expressed genes from the HT were those listed in Flyatlas as being expressed in brain, head, tag, crop, etc and those in the ABD with those expressed in the ovary. (TIF) [file pone.0068136.s001.tif]

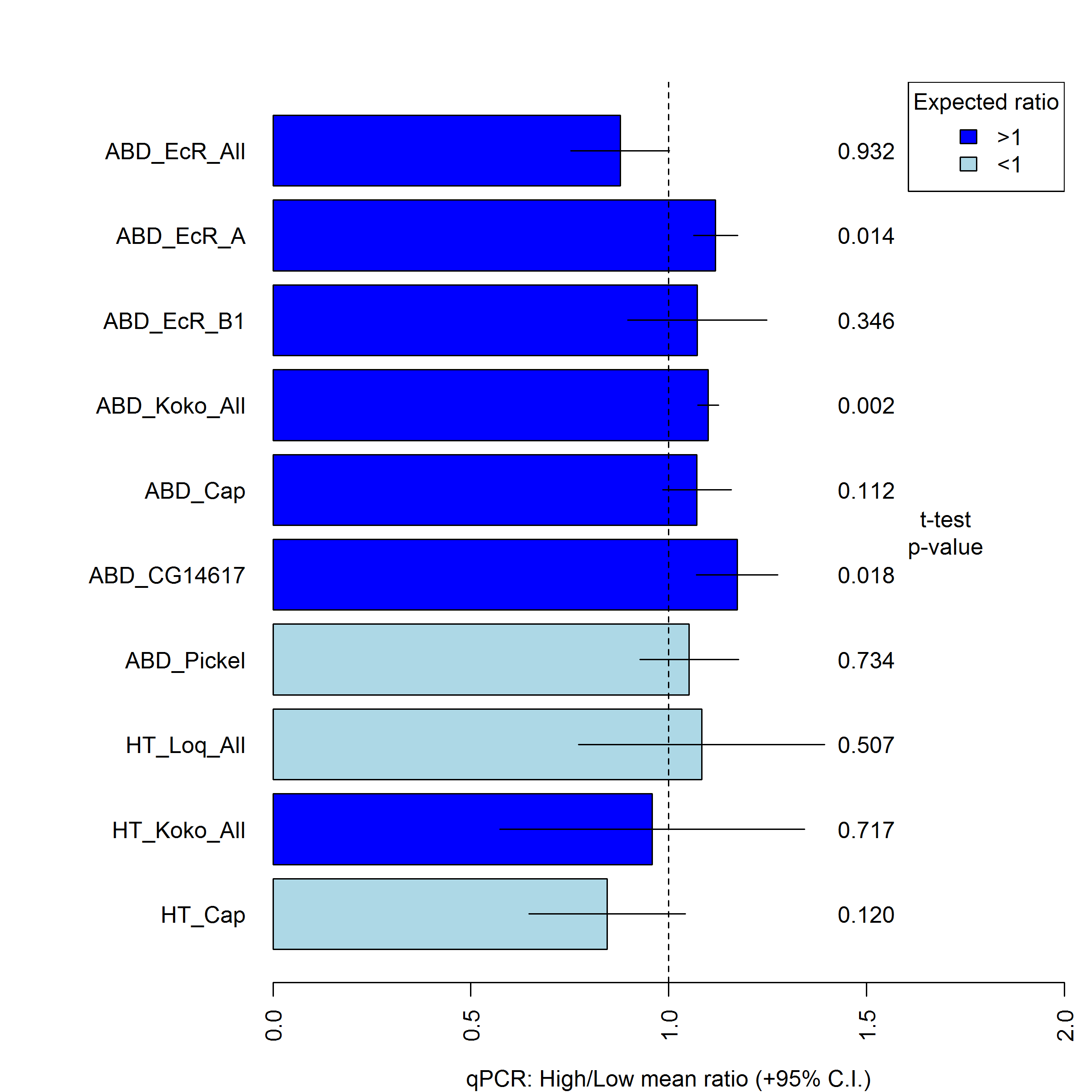

Supplement: Figure S2 — Graphs show the normalised relative levels of gene expression (mean high / low normalised expression ± 95% confidence interval) as determined by qPCR assays for high and low mating treatments in biological replicates 1-4 combined for each of the genes shown. The expected ratio is predicted from the direction of putative differential expression observed in the microarrays (>1 represents putatively up-regulated in the high mating group, and <1 putative down-regulation; see also Table S5). The dotted line represents equal expression in the high and low treatments. One tailed t test P values are shown. (TIFF) [file pone.0068136.s002.tiff]

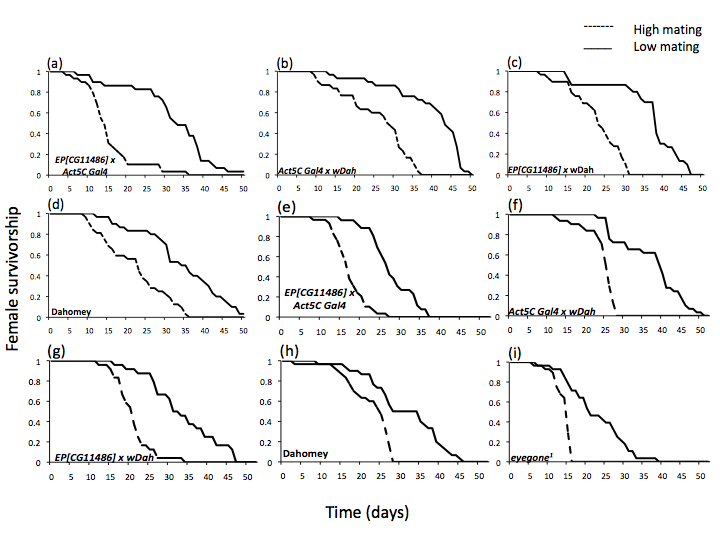

Supplement: Figure S3 — Experiment 1 (a)-(d): survivorship of high and low mating females of the following genotypes: (a) EP[CG11486] x Act5C Gal4 (CG11486 overexpressing), (b) Act5C Gal4 x wDah (control), (c) EP[CG11486] x wDah (control), and (d) Dahomey (control). Experiment 2 (e)-(i): survivorship of high and low mating females of the following genotypes: (e) EP[CG11486] x Act5C Gal4 (CG11486 overexpressing), (f) Act5C Gal4 x wDah (control), (g) EP[CG11486] x wDah (control), (h) Dahomey (control), (i) eyegone 1 (eyegone knockout). (TIF) [file pone.0068136.s003.tif]
