# Supplementary material for: Genome-Wide Responses of Female Fruit Flies Subjected to Divergent Mating Regimes
Source: PLoS One. 2013 Jun 27;8(6):e68136. doi: 10.1371/journal.pone.0068136 (PMC3694895; doi:10.1371/journal.pone.0068136)
Supplement: Table S1 — Sample sizes for the initial tests of mating costs in CG11486 and eyegone manipulated females. (PDF) [file pone.0068136.s004.pdf]

Table S1. Sample sizes for the initial tests of mating costs in *CG11486* and *eyegone* manipulated females in two replicate experiments.

Genotype	Replicate 1		Replicate 2	
	High	Low	High	Low
EP[CG11486] x Act5C Gal4	29	29	29	26
EP[CG11486] x wDah	29	30	24	24
Act5C Gal4 x wDah	30	29	31	29
Dahomey	32	30	30	30
eyegone^{1}	n/a	n/a	28	28
