# Supplementary material for: Genome-Wide Responses of Female Fruit Flies Subjected to Divergent Mating Regimes
Source: PLoS One. 2013 Jun 27;8(6):e68136. doi: 10.1371/journal.pone.0068136 (PMC3694895; doi:10.1371/journal.pone.0068136)
Supplement: Table S2 — (a) Number of females alive per day and timing of sampling for gene expression analysis. (b) Cumulative number of deaths per day. (c) Number of female dead since last censoring. (d) Survivorship (cumulative survival probability). (e) Survivorship differences between treatments (low minus high treatments). (PDF) [file pone.0068136.s005.pdf]

**Table S2(a-e).** Survival data and sample sizes for replicates 1-4 of the experiment to test the effect of exposure to high and low mating treatments on genome-wide gene expression changes in females. (a) Number of females alive per day and timing of sampling for gene expression analysis. (b) Cumulative number of deaths per day. (c) Number of female dead since last censoring. (d) Survivorship (cumulative survival probability). (e) Survivorship differences between treatments (low minus high treatments).

**(a) MORTALITY DATA - NUMBER OF FEMALES ALIVE PER DAY**

| | 40 females sampled for gene expression | | 60 females sampled for gene expression | | | | | |
|---|---|---|---|---|---|---|---|---|
| | REPLICATE 1 | | REPLICATE 2 | | REPLICATE 3 | | REPLICATE 4 | |
| DAY | Dahomey (CONTINUOUS, HIGH MATING) | PoxNeuro[70] (INTERMITTENT, LOW MATING) | Dahomey (CONTINUOUS, HIGH MATING) | PoxNeuro[70] (INTERMITTENT, LOW MATING) | Dahomey (CONTINUOUS, HIGH MATING) | PoxNeuro[70] (INTERMITTENT, LOW MATING) | Dahomey (CONTINUOUS, HIGH MATING) | PoxNeuro[70] (INTERMITTENT, LOW MATING) |
| 1 | 180 | 180 | 180 | 180 | 180 | 180 | 180 | 180 |
| 2 | | | 180 | 180 | 180 | 178 | 180 | 180 |
| 3 | | | | | | | | |
| 4 | | | | | | | | |
| 5 | 176 | 179 | 180 | 178 | 180 | 178 | 180 | 180 |
| 6 | 173 | 179 | 175 | 178 | 178 | 177 | 176 | 177 |
| 7 | | | | | | | | |
| 8 | | | | | | | | |
| 9 | 163 | 177 | 173 | 178 | 168 | 174 | 169 | 175 |
| 10 | 119 | 135 | 113 | 117 | 102 | 111 | 97 | 111 |
| 11 | | | | | | | | |
| 12 | | | | | | | | |
| 13 | 119 | 134 | 109 | 117 | 81 | 109 | 80 | 110 |
| 14 | 118 | 134 | 108 | 116 | 68 | 106 | 79 | 109 |
| 15 | | | | | | | | |
| 16 | | | | | | | | |
| 17 | | | 100 | 114 | 51 | 103 | | |
| 18 | | | 96 | 113 | 39 | 101 | | |
| 19 | | | | | | | | |
| 20 | | | | | | | | |
| 21 | | | 69 | 107 | | | | |
| 22 | | | 55 | 102 | | | | |

## (b) MORTALITY DATA - CUMULATIVE DEATHS PER DAY

| | REPLICATE 1 | | REPLICATE 2 | | REPLICATE 3 | | REPLICATE 4 | |
|---|---|---|---|---|---|---|---|---|
| **DAY** | Dahomey (CONTINUOUS, HIGH MATING) | PoxNeuro[70] (INTERMITTENT, LOW MATING) | Dahomey (CONTINUOUS, HIGH MATING) | PoxNeuro[70] (INTERMITTENT, LOW MATING) | Dahomey (CONTINUOUS, HIGH MATING) | PoxNeuro[70] (INTERMITTENT, LOW MATING) | Dahomey (CONTINUOUS, HIGH MATING) | PoxNeuro[70] (INTERMITTENT, LOW MATING) |
| 1 | 0 | 0 | 0 | 0 | 0 | 0 | 0 | 0 |
| 2 | | | 0 | 0 | 0 | 2 | 0 | 0 |
| 3 | | | | | | | | |
| 4 | | | | | | | | |
| 5 | 4 | 1 | 0 | 2 | 0 | 2 | 0 | 0 |
| 6 | 7 | 1 | 5 | 2 | 2 | 3 | 4 | 3 |
| 7 | | | | | | | | |
| 8 | | | | | | | | |
| 9 | 17 | 3 | 7 | 2 | 12 | 6 | 11 | 5 |
| 10 | 21 | 5 | 7 | 3 | 18 | 9 | 23 | 9 |
| 11 | | | | | | | | |
| 12 | | | | | | | | |
| 13 | 21 | 6 | 11 | 3 | 39 | 11 | 40 | 10 |
| 14 | 22 | 6 | 12 | 4 | 52 | 14 | 41 | 11 |
| 15 | | | | | | | | |
| 16 | | | | | | | | |
| 17 | | | 20 | 6 | 69 | 17 | | |
| 18 | | | 24 | 7 | 81 | 19 | | |
| 19 | | | | | | | | |
| 20 | | | | | | | | |
| 21 | | | 51 | 13 | | | | |
| 22 | | | 65 | 18 | | | | |

**(c) MORTALITY DATA – NUMBER DEAD SINCE PREVIOUS CENSORING**

| | REPLICATE 1 | | REPLICATE 2 | | REPLICATE 3 | | REPLICATE 4 | |
|---|---|---|---|---|---|---|---|---|
| **DAY** | Dahomey (CONTINUOUS, HIGH MATING) | PoxNeuro[70] (INTERMITTENT, LOW MATING) | Dahomey (CONTINUOUS, HIGH MATING) | PoxNeuro[70] (INTERMITTENT, LOW MATING) | Dahomey (CONTINUOUS, HIGH MATING) | PoxNeuro[70] (INTERMITTENT, LOW MATING) | Dahomey (CONTINUOUS, HIGH MATING) | PoxNeuro[70] (INTERMITTENT, LOW MATING) |
| 1 | | | | | | | | |
| 2 | 0 | 0 | 0 | 0 | 0 | 2 | 0 | 0 |
| 3 | | | | | | | | |
| 4 | | | | | | | | |
| 5 | 4 | 1 | 0 | 2 | 0 | 0 | 0 | 0 |
| 6 | 3 | 0 | 5 | 0 | 2 | 1 | 4 | 3 |
| 7 | | | | | | | | |
| 8 | | | | | | | | |
| 9 | 10 | 2 | 2 | 0 | 10 | 3 | 7 | 2 |
| 10 | 4 | 2 | 0 | 1 | 6 | 3 | 12 | 4 |
| 11 | | | | | | | | |
| 12 | | | | | | | | |
| 13 | 0 | 1 | 4 | 0 | 21 | 2 | 17 | 1 |
| 14 | 1 | 0 | 1 | 1 | 13 | 3 | 1 | 1 |
| 15 | | | | | | | | |
| 16 | | | | | | | | |
| 17 | | | 8 | 2 | 17 | 3 | | |
| 18 | | | 4 | 1 | 12 | 2 | | |
| 19 | | | | | | | | |
| 20 | | | | | | | | |
| 21 | | | 27 | 6 | | | | |
| 22 | | | 14 | 5 | | | | |
| **surviving** | 118 | 134 | 55 | 102 | 39 | 101 | 79 | 109 |
| **start** | 140 | 140 | 120 | 120 | 120 | 120 | 120 | 120 |

## (d) SURVIVORSHIP DATA – CUMULATIVE SURVIVAL PROBABILITY

| | REPLICATE 1 | | REPLICATE 2 | | REPLICATE 3 | | REPLICATE 4 | |
|---|---|---|---|---|---|---|---|---|
| DAY | Dahomey (CONTINUOUS, HIGH MATING) | PoxNeuro[70] (INTERMITTENT, LOW MATING) | Dahomey (CONTINUOUS, HIGH MATING) | PoxNeuro[70] (INTERMITTENT, LOW MATING) | Dahomey (CONTINUOUS, HIGH MATING) | PoxNeuro[70] (INTERMITTENT, LOW MATING) | Dahomey (CONTINUOUS, HIGH MATING) | PoxNeuro[70] (INTERMITTENT, LOW MATING) |
| 1 | 1.000 | 1.000 | 1.000 | 1.000 | 1.000 | 1.000 | 1.000 | 1.000 |
| 2 | | | 1.000 | 1.000 | 1.000 | 0.989 | 1.000 | 1.000 |
| 3 | | | | | | | | |
| 4 | | | | | | | | |
| 5 | 0.978 | 0.994 | 1.000 | 0.989 | 1.000 | 0.989 | 1.000 | 1.000 |
| 6 | 0.961 | 0.994 | 0.972 | 0.989 | 0.989 | 0.983 | 0.978 | 0.983 |
| 7 | | | | | | | | |
| 8 | | | | | | | | |
| 9 | 0.906 | 0.983 | 0.961 | 0.989 | 0.933 | 0.967 | 0.939 | 0.972 |
| 10 | 0.850 | 0.964 | 0.942 | 0.975 | 0.850 | 0.925 | 0.808 | 0.925 |
| 11 | | | | | | | | |
| 12 | | | | | | | | |
| 13 | 0.850 | 0.957 | 0.908 | 0.975 | 0.675 | 0.908 | 0.667 | 0.917 |
| 14 | 0.843 | 0.957 | 0.900 | 0.967 | 0.567 | 0.883 | 0.658 | 0.908 |
| 15 | | | | | | | | |
| 16 | | | | | | | | |
| 17 | | | 0.833 | 0.950 | 0.425 | 0.858 | | |
| 18 | | | 0.800 | 0.942 | 0.325 | 0.842 | | |
| 19 | | | | | | | | |
| 20 | | | | | | | | |
| 21 | | | 0.575 | 0.892 | | | | |
| 22 | | | 0.458 | 0.850 | | | | |

## (e) SURVIVORSHIP DATA - TREATMENT DIFFERENCES

| DAY | REPLICATE 1 | REPLICATE 2 | REPLICATE 3 | REPLICATE 4 |
|---|---|---|---|---|
| 0 | 0.000 | 0.000 | 0.000 | 0.000 |
| 1 | 0.000 | 0.000 | 0.000 | 0.000 |
| 2 | 0.000 | 0.000 | -0.011 | 0.000 |
| 5 | 0.017 | -0.011 | -0.011 | 0.000 |
| 6 | 0.033 | 0.017 | -0.006 | 0.006 |
| 9 | 0.078 | 0.028 | 0.033 | 0.033 |
| 10 | 0.114 | 0.033 | 0.075 | 0.117 |
| 13 | 0.107 | 0.067 | 0.233 | 0.250 |
| 14 | 0.114 | 0.067 | 0.317 | 0.250 |
| 17 | | 0.117 | 0.433 | |
| 18 | | 0.142 | 0.517 | |
| 21 | | 0.317 | | |
| 22 | | 0.392 | | |
