# Supplementary material for: Genome-Wide Responses of Female Fruit Flies Subjected to Divergent Mating Regimes
Source: PLoS One. 2013 Jun 27;8(6):e68136. doi: 10.1371/journal.pone.0068136 (PMC3694895; doi:10.1371/journal.pone.0068136)
Supplement: Table S3 — Results are from the RankProd analysis using a pfp<0.05 (see main text for more details). Genes in bold appear in more than one list. (PDF) [file pone.0068136.s006.pdf]

Table S3. All differentially expressed genes in the ABD and HT body parts of high and low mating treatment females. Results are from the RankProd analysis using a pfp<0.05 (see main text for more details). Genes in bold appear in more than one list.

Difference	Transcript	Name/Symbol	Fold Change	pfp	Fs (ANOVA)
ABD down	CG32452-RA	CG32452	0.1498	0	0.0001
	CG14779-RA	pickel	0.1947	0	0.0004
	CG3082-RA	lethal (2) k09913	0.1554	0	0.0005
	**CG10200-RA**	**CG10200**	0.3341	0.004	0.0032
	CG3321-RA	CG3321	0.3368	0.005	0.0050
	CG31012-RA	CG31012	0.3392	0.005	0.0099
	CG32490-RA	complexin	0.3281	0.0057	0.0039
	CG33861-RA	His1:CG33861	0.4522	0.0344	0.0049
	CG1116-RB	CG1116	0.458	0.0387	0.0614
	CG17800-RV	Down syndrome cell adhesion molecule	0.4342	0.0393	0.0076
	CG12484-RB	CG12484	0.4232	0.0393	0.0315
	CG7263-RA	CG7263	0.4555	0.0415	0.0105
	CG32803-RA	CG32803	0.441	0.0433	0.0135
	CG30193-RE	CG30193	0.4548	0.044	0.0008
	CG4912-RA	eEF1delta	0.4733	0.0471	0.0013
	CG33695-RE	CG33695	0.43	0.0473	0.0334
	CG8348-RA	Diuretic hormone	0.4733	0.0475	0.0044
ABD up	CG10488-RA	eyegone	5.6291	0	0.0002
	**CG14617-RC**	**CG14617**	6.5655	0	0.0003
	CG10365-RC	CG10365	5.6804	0	0.0004
	CG10188-RA	CG10188	4.2421	0	0.0005
	**CG3962-RA**	**Keap1**	4.581	0	0.0013
	CG32490-RK	complexin	3.2404	0.0037	0.0009
	CG4881-RA	spalt-related	3.3433	0.0043	0.0027
	**CG11486-RC**	**CG11486**	3.3978	0.005	0.0004
	**CG9802-RA**	**Chromosome-associated protein**	3.3562	0.005	0.0032
	CG1712-RA	Gustatory receptor 43a	2.9704	0.0056	0.0219
	CG2098-RB	ferrochelatase	3.0159	0.0058	0.0122
	CG3653-RA	kin of irre	3.4866	0.0064	0.0079
	CG12943-RA	CG12943	2.8848	0.0069	0.0008
	CG3588-RA	CG3588	2.9119	0.0081	0.0021
	CG11120-RA	CG11120	2.9196	0.0086	0.0044
	CG1148-RA	Osiris 2	2.8489	0.0087	0.0024
	CG33156-RA	CG33156	2.6564	0.0176	0.0070
	CG31000-RC	hephaestus	2.3514	0.025	0.0113
	CG6726-RA	CG6726	2.5691	0.0285	0.0043
	CG17450-RB	CG17450	2.3227	0.0289	0.0038
	CG12822-RA	CG12822	2.2813	0.0319	0.0598
	CG1220-RC	Kaz1-ORFB	2.2126	0.0341	0.0078
	CG14269-RA	CG14269	2.0559	0.0352	0.0050
	CG6416-RG	CG6416	2.0844	0.0362	0.0012
	**CG9147-RA**	**CG9147**	2.4	0.0388	0.0098
	CG13491-RA	Gustatory receptor 58c	2.0257	0.04	0.0025
	CG4878-RA	eIF3-S9	2.341	0.0407	0.0260

	CG5522-RC	CG5522	2.1184	0.0422	0.0323
HT down	**CG9802-RA**	**Chromosome-associated protein**	0.1726	0	6.34E-05
	CG4949-RA	CG4949	0.2234	0	0.0005
	**CG9147-RA**	**CG9147**	0.1327	0	0.0007
	CG8502-RA	Cuticular protein 49Ac	0.2109	0	0.0008
	CG6866-RB	loquacious	0.2186	0	0.0020
	CG32813-RB	CG32813	0.3025	0.0033	0.0024
	CG8068-RC	Suppressor of variegation 2-10	0.258	0.0043	0.0066
	CG13373-RA	CG13373	0.3179	0.0063	0.0008
	**CG3962-RA**	**Keap1**	0.3762	0.0133	0.0150
	CG6921-RA	CG6921	0.3808	0.0138	0.0020
	CG32473-RA	CG32473	0.3701	0.0144	0.0042
	CG12763-RA	Diptericin	0.4261	0.0144	0.0161
	CG34178-RA	-	0.348	0.0145	0.0052
	CG32063-RA	CG32063	0.365	0.015	0.0345
	CG5921-RA	CG5921	0.355	0.0153	0.0043
	CG3056-RA	CG3056	0.3058	0.016	0.0034
	CG15096-RA	CG15096	0.3809	0.0182	0.0032
	CG10371-RA	PTEN-like phosphatase	0.3835	0.0228	0.0044
	CG17800-RAQ	Down syndrome cell adhesion molecule	0.3979	0.0242	0.0153
	CG7757-RA	CG7757	0.3628	0.0285	0.0062
	CG17299-RE	SNF4/AMP-activated protein kinase gamma subunit	0.4492	0.0343	0.0289
	CG8234-RA	CG8234	0.4332	0.0368	0.0738
	CG12006-RA	CG12006	0.3985	0.0387	0.0345
	CG9374-RH	lkb1	0.314	0.0436	0.0075
	**CG10200-RA**	**CG10200**	0.3857	0.0442	0.0099
	**CG11486-RC**	**CG11486**	0.4541	0.0462	0.0018
	CG8660-RB	Fibp	0.434	0.0477	0.0264
	CG1803-RA	regucalcin	0.4617	0.0479	0.0285
HT up	CG1691-RA	IGF-II mRNA-binding protein	4.5009	0	0.0011
	CG31232-RD	kokopelli	3.2792	0.01	0.0020
	CG8009-RA	CG8009	4.2475	0.01	0.0023
	CG6030-RA	ATP synthase, subunit d	3.7959	0.01	0.0038
	CG1891-RA	saxophone	3.7027	0.0125	0.0067
	CG12223-RB	Dorsal switch protein 1	2.6109	0.02	0.0341
	CG9248-RA	CG9248	3.5546	0.0288	0.0074
	CG2368-RF	pipsqueak	2.4868	0.0329	0.0257
	CG6563-RA	Arginine methyltransferase 3	2.7333	0.0392	0.0146
	CG15920-RA	resilin	2.3385	0.04	0.0038
	CG5730-RC	Annexin IX	2.7647	0.0422	0.0086
	CG18787-RA	CG18787	2.3554	0.0423	0.0009
	CG34402-RC	-	2.675	0.0427	0.0027
	CG31219-RA	CG31219	2.2649	0.0467	0.0081
