# Supplementary material for: Genome-Wide Responses of Female Fruit Flies Subjected to Divergent Mating Regimes
Source: PLoS One. 2013 Jun 27;8(6):e68136. doi: 10.1371/journal.pone.0068136 (PMC3694895; doi:10.1371/journal.pone.0068136)
Supplement: Table S4 — Genes and isoforms surveyed by qPCR. Given are PCR primers used for each of the genes tested and the Universal Probe Library probe used in the qPCR analysis. (PDF) [file pone.0068136.s007.pdf]

Table S4. Genes and isoforms surveyed by qPCR. Given are PCR primers used for each of the genes tested and the Universal Probe Library (UPL) probe used in the qPCR analysis.

Gene	Isoform(s)	L-primer	R-primer	UPL
EcR	A,B1,B2	ttaatttggtaccaggatggcta	gttctcatcgggttgactcat	46
EcR	A only	aggttgcagcataggagtcttc	tgtccactcgtcgtcaacat	105
EcR	B1 only	gcagaactggcgcactatatc	gcggtgttttgtgcagttt	22
Kokopelli	A-E	agttgaacaaggcgcagac	gcagcgcactcaaagatgta	165
Loquacious	A,B,C	gtcggtgactgggctcac	ccgttgcatgcacatttc	164
Cap	A,B	tccgggaagagcaacttct	agtgggtgaactcatcgctta	158
CG14617	A-E	caccaaaaagcgcctcac	cttcctgtttcagctcgacat	28
pickel	A,C,D (all)	tcgaaaagggcgactatcc	agctggcgaatgtaactatgg	49
alphaTub84B	A	acacttccaataaaaactcaatatgc	ccgtgctccaagcagtaga	3
