# Supplementary material for: Genome-Wide Responses of Female Fruit Flies Subjected to Divergent Mating Regimes
Source: PLoS One. 2013 Jun 27;8(6):e68136. doi: 10.1371/journal.pone.0068136 (PMC3694895; doi:10.1371/journal.pone.0068136)
Supplement: Table S5 — Shown are (a) the fold changes and significance values from the microrarray analysis, and (b) the isoforms tested by qPCR, the fold difference (high / low treatment expression values), the Universal Probe Library (UPL) probe used and finally significance values from 1 tailed paired t-tests (P values < 0.05 are shown in bold). See also Figure S2. (PDF) [file pone.0068136.s008.pdf]

Table S5. qPCR analysis of candidate DE genes from the microarray analysis. Shown are (a) the fold changes and significance values from the microrarray analysis, and (b) the isoforms tested by qPCR, the fold difference (high / low treatment expression values), the Universal Probe Library (UPL) probe used and finally significance values from 1 tailed paired t tests (*P* values<0.05 are shown in bold). See also Figure S2.

	**(a) Microarray**				**(b) qPCR**			
**Tissue**	**Gene**	**Fold Change**	**ANOVA (*P*-value)**	**RankProd (pfp)**	**Isoforms**	**UPL**	**High/ Low**	***t*-test**
ABD	EcR	0.77	0.000705	0.194	A,B1,B2	46	0.873	0.932
					A only	105	1.116	**0.014**
					B1 only	22	1.042	0.346
	Koko	0.43	0.052212	0.474	A,B,C,D,E	165	1.098	**0.002**
	Cap	1.75	0.003152	0.005	A	158	1.068	0.112
	CG14617	2.71	0.000302	0.000	A,B,C,D,E	28	1.164	**0.018**
	pickel	-2.36	0.00036	0.000	A,C,D (all)	49	1.045	0.734
HT	Koko	1.71	0.001977	0.010	A,B,C,D,E	165	0.829	0.717
	Loq	-2.19	0.002032	0.000	A,B,C	164	1.003	0.507
	Cap	-2.53	6.34E-05	0.000	A	158	0.797	0.120
	CG14617	1.35	0.007136	0.043	A,B,C,D,E	28	failed assay	
