# Supplementary material for: Genome-Wide Responses of Female Fruit Flies Subjected to Divergent Mating Regimes
Source: PLoS One. 2013 Jun 27;8(6):e68136. doi: 10.1371/journal.pone.0068136 (PMC3694895; doi:10.1371/journal.pone.0068136)
Supplement: Table S6 — Enriched functional categories resulting from DAVID/EASE analysis of all differentially expressed genes in the ABD and HT body parts of females subjected to high and low mating treatments (see main text for more details). (PDF) [file pone.0068136.s009.pdf]

Table S6. Enriched functional categories resulting from DAVID/EASE analysis of all differentially expressed genes in the ABD and HT body parts of females subjected to high and low mating treatments (see main text for more details).

Functional Category	Enrichment	Generated by:
intrinsic to plasma membrane	4.02	5 genes
sugar or cation membrane transporters	3.18	18 genes
gustatory/odorant receptors	2.85	35 genes
ion channel activity	2.44	25 genes
