# Supplementary material for: Genome-Wide Responses of Female Fruit Flies Subjected to Divergent Mating Regimes
Source: PLoS One. 2013 Jun 27;8(6):e68136. doi: 10.1371/journal.pone.0068136 (PMC3694895; doi:10.1371/journal.pone.0068136)
Supplement: Table S7 — Mean ± 95% confidence intervals of female survival in days in the initial tests of mating costs in CG11486 and eyegone manipulated females. (PDF) [file pone.0068136.s010.pdf]

Table S7. Mean ± 95% confidence intervals of female survival in days in the initial tests of mating costs in *CG11486* and *eyegone* manipulated females.

Genotype	Replicate 1		Replicate 2	
	**High**	**Low**	**High**	**Low**
EP[CG11486] x Act5C Gal4	15.35 (12.79 - 17.90)	31.93 (27.91 - 35.95)	16.10 (14.60 - 17.61)	26.50 (24.24 - 28.76)
EP[CG11486] x wDah	22.76 (20.27 - 25.25)	36.30 (32.83 - 39.77)	19.75 (17.82 - 21.68)	32.42 (28.64 - 36.20)
Act5C Gal4 x wDah	25.50 (22.19 - 28.81)	39.66 (35.87 - 43.44)	23.29 (21.77 -24.81)	36.10 (32.92 - 39.29)
Dahomey	21.91 (18.85 - 24.96)	34.23 (30.26 - 38.20)	21.50 (19.44 - 23.56)	29.97 (26.22 - 33.71)
eyegone^{1}	N/A	N/A	13.04 (12.14 -13.93)	21.50 (18.46 - 24.54)
